# Supplementary material for: Improving oligo-conjugated antibody signal in multimodal single-cell analysis
Source: eLife. 2021 Apr 16;10:e61973. doi: 10.7554/eLife.61973 (PMC8051954; doi:10.7554/eLife.61973)
Supplement: Supplementary file 1. — Also contains individual clones and concentrations used for the different conditions included in the study. [file elife-61973-supp1.docx]

### Supplementary File 1: Oligo-conjugated antibody panel for 5’-CITE-seq

| **Marker** | **Alias** | **Clone** | **Isotype**  **(Mouse)** | **Vendor** | **TotalSeq C**  **Tag** | **Cat#** | **DF1 conc. (µg/mL)** | **DF4 conc. (µg/mL)** | **Adj. conc. (µg/mL)** | **Adj. conc. (DF1 FC )** |
| --- | --- | --- | --- | --- | --- | --- | --- | --- | --- | --- |
| **CD44** |  | BJ18 | IgG1 | BioLegend | 0125 | 338827 | 0.050 | 0.013 | 0.025 | 2.00 |
| **CD2** |  | TS1/8 | IgG1 | BioLegend | 0367 | 309231 | 0.100 | 0.025 | 0.100 | 1.00 |
| **CD31** | PECAM-1 | WM59 | IgG1 | BioLegend | 0124 | 303139 | 0.100 | 0.025 | 0.100 | 1.00 |
| **CD45** |  | HI30 | IgG1 | BioLegend | 0391 | 304068 | 0.100 | 0.025 | 0.100 | 1.00 |
| **CD123** |  | 6H6 | IgG1 | BioLegend | 0064 | 306045 | 0.500 | 0.125 | 0.500 | 1.00 |
| **CD3** |  | UCHT1 | IgG1 | BioLegend | 0034 | 300479 | 0.500 | 0.125 | 0.500 | 1.00 |
| **CD1c** |  | L161 | IgG1 | BioLegend | 0160 | 331547 | 0.500 | 0.125 | 1.000 | 0.50 |
| **EpCAM** |  | 9C4 | IgG2b | BioLegend | 0123 | 324247 | 0.625 | 0.156 | 0.063 | 10.00 |
| **CD8** |  | RPA-T8 | IgG1 | BioLegend | 0080 | 301071 | 0.625 | 0.156 | 0.078 | 8.00 |
| **CD11b** |  | ICRF44 | IgG1 | BioLegend | 0161 | 301359 | 0.625 | 0.156 | 0.125 | 5.00 |
| **CD5** |  | UCHT2 | IgG1 | BioLegend | 0138 | 300637 | 0.625 | 0.156 | 0.156 | 4.00 |
| **CD69** |  | FN50 | IgG1 | BioLegend | 0146 | 310951 | 0.625 | 0.156 | 0.156 | 4.00 |
| **CD39** |  | A1 | IgG1 | BioLegend | 0176 | 328237 | 0.625 | 0.156 | 0.625 | 1.00 |
| **CD45RA** |  | HI100 | IgG2b | BioLegend | 0063 | 304163 | 0.625 | 0.156 | 0.625 | 1.00 |
| **CD70** |  | 113-16 | IgG1 | BioLegend | 0027 | 355119 | 0.625 | 0.156 | 1.250 | 0.50 |
| **CD196** | CCR6 | G034E3 | IgG2b | BioLegend | 0143 | 353440 | 0.625 | 0.156 | 3.125 | 0.20 |
| **CD19** |  | HIB19 | IgG1 | BioLegend | 0050 | 302265 | 1.000 | 0.250 | 0.100 | 10.00 |
| **HLA-ABC** |  | W6/32 | IgG2a | BioLegend | 0058 | 311449 | 1.000 | 0.250 | 0.125 | 8.00 |
| **CD4** |  | RPA-T4 | IgG1 | BioLegend | 0072 | 300567 | 1.000 | 0.250 | 0.500 | 2.00 |
| **CD14** |  | M5E2 | IgG2a | BioLegend | 0081 | 301859 | 1.000 | 0.250 | 2.000 | 0.50 |
| **CD66b** |  | 6/40c | IgG1 | BioLegend | 0166 | 392909 | 1.000 | 0.250 | 2.000 | 0.50 |
| **CD223** |  | 11C3C65 | IgG1 | BioLegend | 0152 | 369335 | 1.000 | 0.250 | 4.000 | 0.25 |
| **CD56** |  | 5.1H11 | IgG1 | BioLegend | 0047 | 362559 | 1.000 | 0.250 | 4.000 | 0.25 |
| **CD103** |  | BerACT8 | IgG1 | BioLegend | 0145 | 350233 | 1.250 | 0.313 | 0.125 | 10.00 |
| **CD62L** |  | DREG-56 | IgG1 | BioLegend | 0147 | 304851 | 1.250 | 0.313 | 0.125 | 10.00 |
| **TCRab** |  | IP26 | IgG1 | BioLegend | 0224 | 306743 | 1.250 | 0.313 | 0.625 | 2.00 |
| **CD194** | CCR4 | L291H4 | IgG1 | BioLegend | 0071 | 359425 | 1.250 | 0.313 | 1.250 | 1.00 |
| **CD26** | DPP4 | BA5b | IgG2a | BioLegend | 0396 | 302722 | 1.250 | 0.313 | 1.250 | 1.00 |
| **CD274** | B7-H1 (PD-L1) | 29E.2A3 | IgG2a | BioLegend | 0007 | 329751 | 1.250 | 0.313 | 1.250 | 1.00 |
| **CD28** |  | CD28.2 | IgG1 | BioLegend | 0386 | 302963 | 1.250 | 0.313 | 1.250 | 1.00 |
| **CD127** | IL7Ralpha | A019D5 | IgG1 | BioLegend | 0390 | 351356 | 1.250 | 0.313 | 2.500 | 0.50 |
| **CD141** | Thrombomodulin | M80 | IgG1 | BioLegend | 0163 | 344125 | 1.250 | 0.313 | 2.500 | 0.50 |
| **CD25** |  | BC96 | IgG1 | BioLegend | 0085 | 302649 | 1.250 | 0.313 | 2.500 | 0.50 |
| **CD45RO** |  | UCHL1 | IgG2a | BioLegend | 0087 | 304259 | 1.250 | 0.313 | 2.500 | 0.50 |
| **CD80** | B7-1 | 2D10 | IgG1 | BioLegend | 0005 | 305243 | 1.250 | 0.313 | 2.500 | 0.50 |
| **HLA-DR** |  | L243 | IgG2a | BioLegend | 0159 | 307663 | 1.650 | 0.413 | 0.330 | 5.00 |
| **CD117** | C-kit | 104D2 | IgG1 | BioLegend | 0061 | 313243 | 2.500 | 0.625 | 0.625 | 4.00 |
| **IsoIgG1** |  | MOPC-21 |  | BioLegend | 0090 | 400187 | 2.500 | 0.625 | 1.000 | 2.50 |
| **IsoIgG2A** |  | MOPC-173 |  | BioLegend | 0091 | 400293 | 2.500 | 0.625 | 1.000 | 2.50 |
| **CD107a** | LAMP1 | H4A3 | IgG1 | BioLegend | 0155 | 328649 | 2.500 | 0.625 | 2.500 | 1.00 |
| **CD366** |  | F38-2E2 | IgG1 | BioLegend | 0169 | 345049 | 2.500 | 0.625 | 2.500 | 1.00 |
| **CD138** |  | DL-101 | IgG1 | BioLegend | 0831 | 352327 | 3.000 | 0.750 | 3.000 | 1.00 |
| **CD24** |  | ML5 | IgG2a | BioLegend | 0180 | 311143 | 3.300 | 0.825 | 3.300 | 1.00 |
| **CD134** | OX40 | Ber-ACT35 | IgG1 | BioLegend | 0158 | 350035 | 5.000 | 1.250 | 1.000 | 5.00 |
| **CD1a** |  | HI149 | IgG1 | BioLegend | 0402 | 300135 | 5.000 | 1.250 | 1.000 | 5.00 |
| **CD279** | PD-1 | EH12.2H7 | IgG1 | BioLegend | 0088 | 329963 | 5.000 | 1.250 | 1.000 | 5.00 |
| **CD30** |  | BY88 | IgG1 | BioLegend | 0028 | 333919 | 5.000 | 1.250 | 1.000 | 5.00 |
| **CD183** | CXCR3 | G025H7 | IgG1 | BioLegend | 0140 | 353747 | 10.000 | 2.500 | 0.500 | 20.00 |
| **TCRgd** |  | B1 | IgG1 | BioLegend | 0139 | 331231 | 10.000 | 2.500 | 0.500 | 20.00 |
| **CD86** | B7-2 | IT2.2 | IgG2b | BioLegend | 0006 | 305447 | 10.000 | 2.500 | 0.667 | 15.00 |
| **CD152** | CTLA4 | BNI3 | IgG2a | BioLegend | 0151 | 369621 | 10.000 | 2.500 | 1.000 | 10.00 |
| **CD197** | CCR7 | G043H7 | IgG2a | BioLegend | 0148 | 353251 | 10.000 | 2.500 | 1.000 | 10.00 |
