# Supplementary material for: Improving oligo-conjugated antibody signal in multimodal single-cell analysis
Source: eLife. 2021 Apr 16;10:e61973. doi: 10.7554/eLife.61973 (PMC8051954; doi:10.7554/eLife.61973)
Supplement: Supplementary file 2. — Antibody costs of the 52 antibody panel using vendor recommendations for staining volume and concentrations, pre-titration (dilution factor 1) concentrations, and adjusted concentrations. [file elife-61973-supp2.docx]

### Supplementary File 2: Antibody usage and costs

| **Panel data** | **Vendor rec.** | **DF1** | **Adjusted** | **Units** |
| --- | --- | --- | --- | --- |
| **Average Ab conc** | 10.000 | 2.306 | 1.184 | *µg/mL* |
| **Staining volume** | 100 | 50 | 25 | *µl* |
| **Total Ab used*** | 52.000 | 5.996 | 1.539 | *µg* |
| **Cost of Abs per sample**** | 1690 | 194.857 | 50.024 | *USD* |
| **Fold Reductions in cost compared to** |  |  |  |  |
| **Vendor rec.** | 1.0 | 8.7 | 33.8 | *Fold* |
| **DF1** | - | 1.0 | 3.9 | *Fold* |
| **Vendor rec. (same volume)** | 1.0 | 4.3 | 8.4 | *Fold* |
| **DF1 (same volume)** | - | 1.0 | 1.9 | *Fold* |

*The table is based on the 52 marker TotalSeq-C antibody panel shown in Figure 1-Figure Supplement 1

**Price calculations based on USD 325 per 10µg of TotalSeq-C antibody (list price as of 2021-02-23 from BioLegend).
